# Socioeconomic and marital status among liver cirrhosis patients and associations with mortality: a population-based cohort study in Sweden

**DOI:** 10.1186/s12889-020-09783-2

**Published:** 2020-11-30

**Authors:** Juan Vaz, Ulf Strömberg, Berne Eriksson, David Buchebner, Patrik Midlöv

**Affiliations:** 1 Department of Clinical Sciences in Malmö, Center for Primary Health Care Research, Lund University, Malmö, Sweden; 2 Department of Internal Medicine, Halland Hospital Halmstad, Lasarettsvägen, 302 33 Halmstad, Sweden; 3 Institute of Medicine, Sahlgrenska Academy at University of Gothenburg, Gothenburg, Sweden; 4 Department of Research and Development, Region Halland, Halmstad, Sweden; 5 Krefting Research Centre, Institute of Medicine, University of Gothenburg, Gothenburg, Sweden

**Keywords:** Liver cirrhosis, Socioeconomic status, Marital status, Employment, Occupation, Sweden

## Abstract

**Background:**

The importance of socioeconomic status for survival in cirrhosis patients is more or less pronounced within different populations, most likely due to cultural and regional differences combined with dissimilarities in healthcare system organisation and accessibility. Our aim was to study the survival of patients with cirrhosis in a population-based Swedish cohort, using available data on marital status, employment status, and occupational skill level.

**Methods:**

We conducted a retrospective cohort study of 582 patients diagnosed with cirrhosis in the Region of Halland (total population 310,000) between 2011 and 2018. Medical and histopathologic data, obtained from registries, were reviewed. Cox regression models were used to estimate associations between survival and marital status (married, never married, previously married), employment status (employed, pensioner, disability retired, unemployed), and occupational skill level (low-skilled: level I; medium-skilled: level II; medium-high skilled: level III; professionals: level IV); adjusting for sex, age, aetiology, Model for End-stage Liver Disease (MELD) score, Child-Pugh class, and comorbidities.

**Results:**

Alcohol was the most common aetiology (51%). Most patients were male (63%) and the median age was 66 years. Occupational skill level was associated with the severity of cirrhosis at diagnosis and the prevalence of Child-Pugh C gradually increased from professionals through low-skilled. The mean survival for professionals (6.39 years, 95% CI 5.54–7.23) was higher than for low-skilled (3.00 years, 95% CI 2.33–3.67) and medium-skilled (4.04 years, 95% CI 3.64–4.45). The calculated hazard ratios in the multivariate analysis were higher for low-skilled (3.43, 95% CI 1.89–6.23) and medium-skilled (2.48, 95% CI 1.48–4.12), compared to professionals. When aggregated, low- and medium-skilled groups also had poorer mean survival (3.79 years, 95% CI 3.44–4.14; vs 5.64 years, 95% CI 5.00–6.28) and higher hazard ratios (1.85, 95% CI 1.32–2.61) compared to the aggregated medium-high skilled and professional groups. Marital and employment status were not statistically significant predictors of mortality in the multivariate analysis.

**Conclusions:**

Occupational skill level was strongly associated with mean survival and mortality risk. Poorer prognosis among patients with low and medium occupational skill level could not be explained by differences in sex, age, marital status, employment status, MELD score, Child-Pugh class, or comorbidity.

**Supplementary Information:**

The online version contains supplementary material available at 10.1186/s12889-020-09783-2.

## Background

Liver cirrhosis is a major cause of mortality that each year is responsible for over one million deaths worldwide [1]. It is the main risk factor for hepatocellular cancer (HCC) [2], and it is also the leading indicator for liver transplantation in Europe [3]. Alcohol overconsumption and chronic hepatitis C infection (HCV) are the main causes of cirrhosis in Sweden [4–6].

Socioeconomic status (SES) refers to an individual’s position in society; SES is established by a combination of educational, occupational, and economic criteria [7]. SES has previously been associated with mortality and morbidity, regardless of whether it is measured by educational level, occupation or income [8]. Although these are not completely interchangeable, the different measures of SES are related to each other to a large extent [9, 10]. Higher educational level is generally associated with qualified occupations and higher income [9].

Individuals with high SES may report comparable quantities of alcohol intake, or even higher, compared to individuals with lower SES [11]. However, lower SES has been associated with a disproportionate burden of alcohol-related disease [11]. In Sweden, a strong relationship between low SES and the incidence of alcohol-related disease was observed during the end of the past century [12]. Mortality, due to alcohol-related disease, was also higher among lower SES groups between 1991 and 2006 [13]. Globally, low SES has also been associated with increased risk for HCV and with poorer prognosis among these patients [14].

While the associations between SES and increased risk for cirrhosis are being elucidated, less is known regarding the association between SES and survival among cirrhosis patients [15–21]. Divorced patients and patients who were disability retired had poorer survival rates in Denmark [15]. However, personal income was not associated with survival [15]. In Barcelona, Spain, patients with lower educational level and patients living in socioeconomically deprived areas had the highest cirrhosis-related mortality [16]. In Australia, manual workers have been reported to have increased cirrhosis-related mortality rates over time, when compared to non-manual workers [18]. In the USA, racial/ethnic minorities, shorter formal education, and poverty have been reported as important risk factors for increased cirrhosis-related mortality [20, 21].

Personal income level, marital status, employment and occupation appear to be related to overall survival in cirrhosis patients. However, the importance of these factors might be more or less pronounced within different populations. Cultural and regional differences, combined with dissimilarities in healthcare system organisations and accessibility [22], might be of importance concerning the role of SES in cirrhosis survival. To date, no other study has investigated the importance of SES and marital status on cirrhosis mortality among Swedish patients during the last decade (2010–2019). Our primary aim was to study the survival of patients with cirrhosis in a population-based Swedish cohort, using available data on marital status, employment status, and occupational skill level. We also aimed to compare the causes of death between the different patient groups.

## Methods

### Study population and patient data

This study is based on a cohort described earlier in detail [6], which was retrieved by a systematic inventory of cirrhosis data in a well-defined Swedish population. Essentially, a broad search was performed for all patients with cirrhosis diagnosed in the Region of Halland (310,665 inhabitants, year 2014) between January 1st 2011 and December 31st 2018 [6].

The search was conducted using a wide array of cirrhosis-related International Classification of Diseases 10th Revision – Swedish Edition (ICD-10-SE) codes (Supplementary Material 1) [6]. The use of ICD-10-SE has been mandatory in Sweden since January 1st 2011 and, according to the Swedish National Board of Health and Welfare, none of the codes described in the supplementary material have been changed since then [23]. In addition, patient data were retrieved from the pathology registry using the Systemized Nomenclature of Medicine (SNOMED) codes for liver (T-56), cirrhosis (M-495), and HCC (M-817) as described elsewhere [5, 6]. SNOMED is an international nomenclature system that is broadly used in pathology [24].

Liver cirrhosis was diagnosed by histology, or based on clinical and laboratory findings combined with standardised radiological features previously described [6, 25]. All patients without distinctive radiological features or typical histology confirming the diagnosis of cirrhosis, were excluded. Minor patients (age under 18 years), patients not registered as permanent residents in the Region of Halland, and those diagnosed before 2011 or after 2018, were also excluded.

All medical records were thoroughly reviewed and information was retrieved regarding age, sex, date of diagnosis, aetiology, marital status, employment status, occupation, length and weight, complications and comorbidities at diagnosis, laboratory results, use of warfarin, date of moving from the Region of Halland, liver transplantation or death and cause of death.

### Aetiological groups

Each patients was included into one the following aetiological groups: alcohol, HCV, Non-alcoholic fatty liver disease (NAFLD), cryptogenic cirrhosis, primary biliary cholangitis, autoimmune hepatitis, and “Other causes” [6]. “Other causes” comprised patients with less common aetiologies in our cohort (observed in a previous study) [6], such as primary sclerosing cholangitis, hepatitis B and hemochromatosis. Alcohol-related cirrhosis was defined for patients with a history of long-lasting alcohol overconsumption and/or elevated concentrations of phosphatidylethanol, or (in some cases) carbohydrate-deficient transferrin [6]. If a patient had both alcohol overconsumption and HCV as possible causes of cirrhosis, only the latter was registered as the aetiology [6]. Patients without a well-defined cause of cirrhosis were regarded as having cryptogenic cirrhosis. NAFLD was only used when diagnosed by clinicians or verified through biopsy.

### Indicators of socioeconomic status: marital status, employment status, and occupational skill level

In addition to the electronic registries described earlier, medical journal data from scanned reports from 1990 to 2011 were revised when compiling SES. These medical records included data regarding sick-leave certificates, disability certificates and certificates of fitness. These certificates were in turn regulated by the Swedish Social Insurance Agency, and included employment status and main reported occupation. Patients were classified as married (including cohabiting), previously married (separated, divorced or widowed), and never married, at the time of diagnosis. Patients were also classified according to their employment status at the time of diagnosis: employed, pensioner, disability retired, or unemployed. Employment status is strongly associated with the mean disposable annual income (Supplementary Material 2).

Patients’ occupations were categorised according to the Swedish Standard Classification of Occupations 2012 (SSYK 2012) [26]. SSYK 2012 is based on the International Standard Classification of Occupations 2008 (ISCO-08), which grades occupations into four main occupational skill levels (level I-IV) [27]. Skill level refers to type of working task and its complexity. Translated into a Swedish context, level I represents elementary occupations – low-skilled (e.g. cleaners), level II includes most skilled manual-workers – medium-skilled (e.g. plumbers, drivers), level III represents occupations requiring up to 3 years of tertiary education – medium-high skilled (e.g. IT technicians, real estate agents), and level IV mainly stands for managers and professionals – high- and very high skilled [26, 27]. In Sweden, there are strong associations between occupational skill level and educational level (Table 1). In turn, occupational skill level is strongly linked to income level (Supplementary Material 2).

**Table 1:** Associations between SSYK 2012, occupational skill levels (ISCO-08), and the international classification of education ISCED-97

SSYK 2012	ISCO-08 occupational skill level	ISCED-97 level
Elementary occupations	I	Level 1 Elementary education at primary school
Administration and customer service clerks	II	Level 2–4 Education programmes at upper secondary and tertiary level of not more than 2 years in length
Service, care and shop sales workers	II	
Agricultural, horticultural, forestry and fishery workers	II	
Building and manufacturing workers	II	
Mechanical manufacturing and transport workers, etc.	II	
Other ranks (privates. Etc.).	II	
Non-commissioned officers	III	Level 5b Practical or vocational tertiary education programmes of 2–3 years in length
Operations managers in service industries	III	
Occupations requiring higher education qualifications or equivalent	III	
Managers	IV	Level 5a-6 Theoretical or research-oriented tertiary education programmes and third-cycle programmes of at least 3 years, normally 4 years or longer in length
Commissioned officers	IV	
Occupations requiring advanced level of higher education	IV	

ISCED-97: International Standard Classification of Education, UNESCO 1997; ISCO-08: International Standard Classification of Occupations 2008; SSYK 2012: Swedish Standard Classification of Occupations 2012. A more detailed description can be found at Statistics Sweden (www.scb.se)

Each patient (represented once) was included in one of the main occupational skill level groups. If several occupations were registered for a patient, only the most representative (longest period of time or main income source during work-life) was registered. If a patient had various simultaneous occupations, only the one referred by the patient (or the treating physician) as the primary was registered. Pensioners and disability retired were classified according to the main occupation reported during their work-life. Unemployed, without known previous occupation, or with a history of longstanding unemployment (10–15 years prior to cirrhosis diagnosis), were included in the occupational skill level I.

### Comorbidities, complications and severity

A comorbidity was included if diagnosed for up to 10 years prior to, and observed at the time of cirrhosis diagnosis. Body mass index (BMI) values (kg/m^2^) were calculated as defined by WHO [28]. The following definitions were applied: underweight (BMI < 18.5), normal weight (BMI 18.5–24.9), pre-obesity (BMI 25.0–29.9), and obesity (BMI > 29.9) [28]. Ascites was registered if detected clinically and/or radiologically. Variceal bleeding was assumed upon evident signs of bleeding according to the Baveno IV classification of significant bleeding [29]. Hepatic encephalopathy was registered if observed at diagnosis or under the initial follow-up. Only HCC cases diagnosed at the time of cirrhosis diagnosis, or within the 6 months after, were considered a complication.

Model of end-stage liver disease (MELD) scores and Child-Pugh class were calculated and used as indicators of cirrhosis severity [30, 31]. Patients were divided into the following groups: MELD < 10, MELD 10–14, and MELD ≥15. Patients with advanced chronic kidney disease and/or treatment with warfarin were excluded from the calculation of MELD score and Child-Pugh class, being thereby considered as missing data for these variables.

### Follow-up and end-point data

Each patient was followed until December 31st 2019, date of transplantation, death or moving from the Region of Halland (whichever occurred first). End-point data were automatically linked to our medical record system via the Swedish Civil Registration System. For deceased patients, the cause of death was obtained from our medical system as all death certificates for residents in the Region of Halland are routinely scanned and integrated into the medical journal. These death certificates are duplicates of the ones sent to the Swedish National Board of Health and Welfare. If the cause of death was missing, the data were obtained from the Swedish National Cause of Death Register.

### Statistical analysis

Data were expressed as medians and percentiles, or as numbers and percentages, depending on the type of variable presented. Chi-square test, or Fisher’s test (when appropriate), was performed for the comparison of sex, aetiology, comorbidities, stratified MELD score, Child-Pugh class, and complications between the different marital status, employment status, and occupational skill levels. Median values for age at diagnosis and MELD score were compared among the different marital status, employment status, and occupational skill levels, by the Kruskal-Wallis test [32]. Chi-square test was used for the comparison of causes of death between the different sexes, marital status, employment, and occupational skill level. Missing data was expressed as percentages.

Primarily, we considered transplant-free survival, i.e. each patient was followed from date of diagnosis until date of death, transplant surgery, moving from the Region of Halland, or December 31st 2019, whichever occurred first. Moreover, we analysed survival among the patients who underwent a transplant, i.e. each such patient was followed from date of transplant surgery until date of death or December 31st 2019, whichever occurred first. Kaplan-Meier estimates with Greenwood confidence intervals (CI) were used to determine mean survival [33]. Survival curves were compared using the log-rank test. Cox regression models were used to determine hazard ratios (HRs) for the variables of interest: sex, age, marital status, employment status, occupational skill level, aetiology, MELD score, and Child-Pugh class at diagnosis. For a variable with > 2 categories, a reference category was selected and the HRs corresponding to each other category were estimated. Unadjusted HRs were estimated from univariate Cox models, while the adjusted HRs were obtained from a multivariate Cox model that included all variables, together with the registered comorbidities, as covariates. A *p*-value < 5% from a two-tailed test was referred to as statistically significant. All tests were conducted using IBM SPSS Statistics for Macintosh (version 26.0, IBM Statistics, Amorak, NY, USA).

## Results

### Study population

**Fig. 1:**
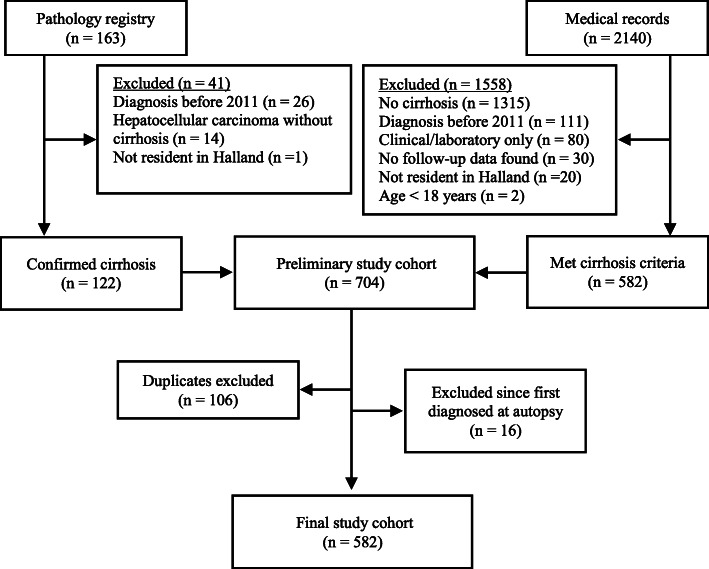
Cirrhosis and socioeconomic status in Halland (Sweden), 2011–2018. Identification Flowchart (This figure has been modified after Vaz et al. [6])

A total of 598 patients with cirrhosis were identified. From this total, we excluded 16 patients as they were not diagnosed with cirrhosis before autopsy. The final study cohort comprised 582 patients (Fig. 1). Most patients had alcohol-related cirrhosis (51%), and the median age at diagnosis was 66 years. Missing BMI values were registered for 3.3% of the cohort. Normal weight and pre-obesity (35% each) were common, and obesity was registered in 23% of the cohort. Due to missing data, MELD scores and Child-Pugh class were not available for 4.5% of the cohort. The associations between marital status, employment status and occupational skill level are shown in Supplementary Material 3.

### Marital status: associations with aetiology, comorbidity, severity and complications at diagnosis

Previously married individuals had the highest median age at diagnosis (71 years; *p* < 0.0001) (Table 2). There were no statistically significant differences regarding the aetiology of cirrhosis upon marital status (*p* = 0.083). Arterial hypertension and diabetes mellitus were more common among the married individuals (*p* = 0.047 and 0.025, respectively). The never married group had the highest rate of obesity (*p* = 0.010) and MELD ≥15 at diagnosis (*p* = 0.041). There were no statistically significant differences within marital status groups regarding median MELD, Child-Pugh class or complications observed at diagnosis.

**Table 2:** Baseline characteristics of the 582 patients diagnosed with cirrhosis. Marital and employment status

	Marital status, n (%)			***P***-value	Employment status, n (%)				***P***-value	Total, n (%)
	Married 339 (58)	Never married 141 (24)	Prev. married 102 (18)	< 0.0001	Employed 171 (29)	Pensioner 273 (47)	Disability retired 93 (16)	Unemployed 45 (8)	< 0.0001	582 (100)
Male	213 (63)	100 (71)	55 (54)	0.025	118 (69)	165 (60)	54 (58)	31 (69)	0.168	368 (63)
Female	126 (37)	41 (29)	47 (46)	0.025	53 (31)	108 (40)	49 (42)	14 (31)	0.168	214 (37)
Median age	65	64	71	< 0.0001	49	72	63	55	< 0.0001	66
(10–90 percentile)	(51–80)	(48–81)	(55–86)		(46–69)	(66–84)	(51–77)	(39–62)		(50–81)
**Aetiologies**										
Alcohol	176 (52)	73 (52)	46 (45)	0.083	96 (56)	130 (48)	47 (51)	22 (49)	< 0.0001	295 (51)
Cryptogenic	50 (15)	17 (12)	16 (16)	0.083	9 (5)	66 (24)	8 (9)	0 (0)	< 0.0001	83 (14)
Hepatitis C	36 (11)	30 (21)	14 (14)	0.083	31 (18)	7 (3)	24 (26)	18 (40)	< 0.0001	80 (14)
PBC	21 (6)	2 (1)	8 (8)	0.083	11 (6)	16 (6)	4 (4)	0 (0)	< 0.0001	31 (5)
NAFLD	16 (5)	9 (6)	4 (4)	0.083	5 (3)	18 (7)	6 (6)	0 (0)	< 0.0001	29 (5)
AIH	18 (5)	5 (4)	7 (7)	0.083	8 (5)	20 (7)	1 (1)	1 (2)	< 0.0001	30 (5)
Other causes	22 (6)	5 (4)	7 (7)	0.083	11 (6)	16 (6)	3 (3)	4 (9)	< 0.0001	34 (6)
**Comorbidities**										
Arterial hypertension	123 (36)	36 (26)	29 (28)	0.047	57 (33)	90 (33)	35 (38)	6 (13)	0.033	188 (32)
CAD	59 (17)	22 (16)	24 (24)	0.254	17 (10)	61 (22)	26 (28)	1 (2)	< 0.0001	105 (18)
Diabetes mellitus	109 (32)	28 (20)	30 (29)	0.025	48 (28)	83 (30)	30 (32)	6 (13)	0.103	167 (29)
Obesity^a^	81 (24)	37 (26)	16 (16)	0.010	46 (27)	55 (20)	23 (25)	10 (22)	0.473	134 (23)
**Severity**^b^										
MELD (median)	11	13	10	0.161	10	11	12	13	0.156	11
< 10	121 (36)	47 (33)	44 (43)	0.041	80 (47)	88 (32)	31 (33)	13 (29)	0.050	212 (36)
10–14	93 (27)	29 (21)	17 (17)	0.041	31 (18)	73 (27)	24 (26)	11 (24)	0.050	139 (24)
≥ 15	108 (32)	61 (43)	36 (35)	0.041	57 (33)	90 (33)	37 (40)	21 (47)	0.050	205 (35)
Child-Pugh class										
A	134 (40)	42 (30)	34 (33)	0.172	82 (48)	80 (29)	37 (40)	11 (24)	0.001	210 (36)
B	125 (37)	58 (41)	41 (40)	0.172	46 (27)	121 (44)	36 (39)	21 (47)	0.001	224 (38)
C	62 (18)	37 (26)	22 (22)	0.172	40 (23)	49 (18)	19 (20)	13 (29)	0.001	121 (21)
**Complications**										
Ascites	151 (45)	73 (52)	51 (50)	0.292	67 (39)	147 (54)	39 (42)	22 (49)	0.016	275 (47)
Variceal bleeding	21 (6)	7 (5)	4 (4)	0.643	10 (6)	14 (5)	4 (4)	4 (9)	0.714	32 (6)
Encephalopathy	18 (5)	15 (11)	10 (10)	0.075	13 (8)	16 (6)	9 (10)	5 (11)	0.463	43 (7)
HCC	45 (13)	18 (13)	11 (11)	0.803	12 (7)	45 (17)	13 (14)	4 (9)	0.026	74 (13)

*AIH* Autoimmune hepatitis; *CAD* Cardiac artery disease; *HCC* Hepatocellular carcinoma; *MELD* Model for end-stage liver disease; *NAFLD* Non-alcoholic fatty liver disease; *PBC* Primary biliary cholangitis. ^a^ Obesity defined as body mass index > 29.9 kg/m^2^, body mass index value calculated for 96.7% of patients. ^b^ MELD-score and Child-Pugh class calculated for 95.5% of patients

### Employment status: associations with aetiology, comorbidity, severity and complications at diagnosis

Employed had the lowest median age at diagnosis (49 years; *p* < 0.0001) (Table 2). These patients also had the highest percent of alcohol-related cirrhosis (*p* < 0.0001). Cryptogenic cirrhosis was common in pensioners (24%), and HCV was very common among the unemployed (40%). Arterial hypertension was uncommon among the unemployed, and cardiac artery disease was more common among the disability retired (*p* = 0.033 and < 0.0001; respectively). Unemployed had the highest rate of Child-Pugh C, at diagnosis (29%; *p* = 0.001). These patients also had the highest median MELD and MELD ≥15 rate at diagnosis, but these associations were not statistically significant (*p* = 0.050, and 0.156; respectively). Employed had the lowest rate of ascites (39%; *p* = 0.016). Pensioners and disability retired had the highest rate of prevalent HCC at diagnosis (17 and 14%, respectively; *p* = 0.026).

### Occupational skill: associations with aetiology, comorbidity, severity and complications at diagnosis

Occupational skill level IV consisted of only 55 patients (9%; *p* < 0.0001), and there was a marked male predominance (80%; *p* = 0.001) (Table 3). Occupational skill level I had the highest frequency of HCV (30%; *p* < 0.0001). Arterial hypertension was lowest in occupational skill level I (16%; *p* < 0.0001), and cardiac artery disease was uncommon in occupational skill level IV (*p* = 0.027). Child-Pugh C was more common in occupational skill level I (28%; *p* = 0.010). Although MELD ≥15 was 2.15 times more frequent in occupational skill level I compared to occupational skill level IV, this association was not statistically significant (*p* = 0.178). The prevalence of complications at diagnosis was significantly different between these two occupational skill levels, as ascites and hepatic encephalopathy were 1.6 and 6 times higher in occupational skill level I (*p* = 0.045 and *p* = 0.022, respectively). There were no statistically significant differences regarding the prevalence of other comorbidities or complications at diagnosis among the different occupational skill levels.

**Table 3:** Baseline characteristics of the 582 patients diagnosed with cirrhosis. Occupational skill level

	Occupational skill level, n (%)				***P***-value
	I 115 (20)	II 348 (60)	III 64 (11)	IV 55 (9)	< 0.0001
Male	63 (55)	230 (66)	31 (48)	44 (80)	0.001
Female	52 (45)	118 (34)	33 (52)	11 (20)	0.001
Median age	63	67	66	65	0.074
(10–90 percentile)	(46–84)	(53–80)	(52–82)	(49–77)	
**Aetiologies**					
Alcohol	44 (38)	187 (54)	36 (56)	28 (51)	< 0.0001
Cryptogenic	14 (12)	53 (15)	11 (17)	5 (9)	< 0.0001
Hepatitis C	34 (30)	36 (10)	3 (5)	7 (13)	< 0.0001
PBC	3 (3)	24 (7)	1 (2)	3 (6)	< 0.0001
NAFLD	5 (4)	19 (6)	3 (5)	2 (4)	< 0.0001
AIH	5 (4)	14 (4)	7 (11)	4 (7)	< 0.0001
Other causes	10 (9)	15 (4)	3 (5)	6 (11)	< 0.0001
**Comorbidities**					
Arterial hypertension	18 (16)	126 (36)	24 (38)	20 (36)	< 0.0001
CAD	15 (13)	75 (22)	11 (17)	4 (7)	0.027
Diabetes mellitus	27 (24)	106 (31)	19 (30)	15 (27)	0.543
Obesity^a^	20 (17)	87 (25)	19 (30)	8 (15)	0.127
**Severity**^b^					
MELD (median)	13	11	11	10	0.223
< 10	35 (30)	134 (39)	22 (34)	21 (38)	0.178
10–14	26 (23)	80 (23)	16 (25)	17 (31)	0.178
≥ 15	50 (43)	121 (35)	23 (36)	11 (20)	0.178
Child-Pugh class					
A	25 (22)	136 (39)	24 (38)	25 (45)	0.010
B	54 (47)	127 (36)	26 (41)	17 (31)	0.010
C	32 (28)	71 (20)	11 (17)	7 (13)	0.010
**Complications**					
Ascites	65 (57)	159 (46)	32 (50)	19 (35)	0.045
Variceal bleeding	9 (8)	19 (6)	4 (6)	0 (0)	0.215
Encephalopathy	14 (12)	27 (8)	1 (2)	1 (2)	0.022
HCC	19 (17)	46 (13)	7 (11)	2 (4)	0.119

*AIH* Autoimmune hepatitis; *CAD* Cardiac artery disease; *HCC* Hepatocellular carcinoma; *MELD* Model for end-stage liver disease; *NAFLD* Non-alcoholic fatty liver disease; *PBC* Primary biliary cholangitis. ^a^ Obesity defined as body mass index > 29.9 kg/m^2^, body mass index value calculated for 96.7% of patients. ^b^ MELD-score and Child-Pugh class calculated for 95.5% of patients

### Transplant-free survival analysis

During an accumulated follow-up time of 1684 person-years, a total of 319 patients (55%) died, including one of the 18 patients who previously underwent a transplant. The follow-up was censored for the remaining 246 patients. The mean follow-up for surviving patients was 4.52 years (range 1.01–8.95), and the mean survival time was 4.41 years (95% CI, 4.08–4.73).

**Fig. 2:**
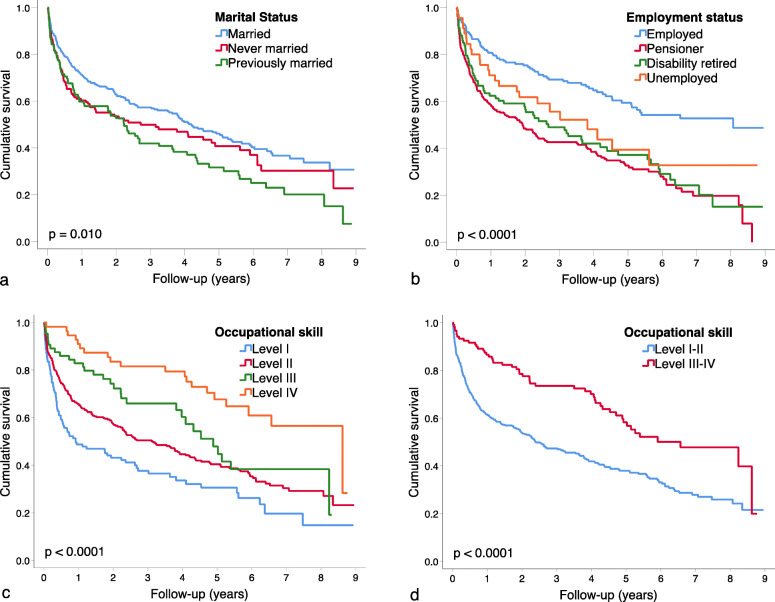
Kaplan-Meier survival curves in the cohort of 582 patients with cirrhosis diagnosed in Halland (2011–2018). Compared by: a) marital status; b) employment status; c) occupational skill level (I through IV); d) occupational skill level (I-II vs III-IV). Occupational skill levels according to the International Standard Classification of Occupations, defined by the International Labour Organisation in 2008 (http://www.ilo.org)

Men had lower mean survival compared to women (3.81 vs 4.82 years; *p* = 0.003). This association was consistent with the calculated HR in the univariate analysis (Table 4). Married patients had better mean survival compared to those previously married (4.56 vs 3.35 years; *p* = 0.010) (Fig. 2). Previously married (HR 1.50, 95% CI 1.14–1.96) had higher HR compared to married individuals in the univariate analysis. However, marital status was not a significant predictor of mortality in the multivariate analysis. Employed had the highest mean survival (5.74 years, 95% CI 5.17–6.32), followed by unemployed (4.30 years, 95% CI 3.15–5.45). There were only minor differences regarding mean survival between disability retired and pensioners (3.63 vs 3.28 years; *p* < 0.0001) (Fig. 2). This association was consistent with the calculated HR in univariate analysis (*p* < 0.0001) (Table 4). However, as with marital status, employment was not a statistically significant predictor for mortality in the multivariate analysis.

**Table 4:** Survival analysis and hazard ratios for 582 patients diagnosed with cirrhosis in Halland 2011–2018

	Mean transplantation-free survival in years (95% CI)	Univariate analyses Hazard ratio (95% CI)	Multivariate analysis 1 Hazard ratio (95% CI)	Multivariate analysis 2 Hazard ratio (95% CI)
**Sex**				
Female	4.82 (4.29–5.35)	1.00 (reference)	1.00 (reference)	1.00 (reference)
Male	3.81 (3.42–4.20)	1.42 (1.12–1.78)	1.37 (1.03–1.81)	1.28 (0.96–1.66)
**Age**				
18–44	6.17 (4.68–7.66)	1.00 (reference)	1.00 (reference)	1.00 (reference)
45–49	6.10 (5.13–7.06)	1.08 (0.41–2.84)	1.55 (0.58–4.17)	1.57 (0.59–4.22)
50–54	5.23 (4.10–6.37)	1.63 (0.69–3.88)	2.70 (1.08–6.75)	2.69 (1.08–6.73)
55–59	5.06 (4.11–6.01)	1.81 (0.80–4.11)	3.73 (1.57–8.83)	3.50 (1.49–8.27)
60–64	4.79 (4.03–5.54)	2.00 (0.91–4.41)	3.85 (1.67–8.85)	3.61 (1.58–8.25)
65–69	4.48 (3.79–5.16)	2.17 (0.99–4.76)	5.67 (2.38–13.5)	5.52 (2.31–13.2)
70–74	3.68 (2.86–4.50)	2.97 (1.34–6.60)	7.75 (3.15–19.0)	7.23 (2.96–17.7)
75–79	2.80 (1.97–3.63)	4.07 (1.83–9.07)	9.60 (3.86–23.8)	9.50 (3.84–23.5)
80+	1.13 (0.80–1.46)	7.97 (3.64–17.5)	14.6 (5.82–36.5)	14.5 (5.79–36.1)
**Socioeconomic and marital status**				
Marital status				
Married	4.56 (4.14–4.98)	1.00 (reference)	1.00 (reference)	1.00 (reference)
Never married	3.97 (3.31–4.63)	1.24 (0.96–1.61)	1.05 (0.78–1.42)	1.06 (0.79–1.44)
Previously married	3.35 (2.69–4.01)	1.50 (1.14–1.96)	1.03 (0.76–1.40)	1.08 (0.80–1.46)
Employment status				
Employed	5.74 (5.17–6.32)	1.00 (reference)	1.00 (reference)	1.00 (reference)
Pensioner	3.28 (2.85–3.70)	2.39 (1.81–3.16)	0.89 (0.60–1.32)	0.88 (0.59–1.30)
Disability retired	3.63 (2.91–4.35)	2.10 (1.49–2.95)	1.44 (0.96–2.16)	1.54 (0.99–2.24)
Unemployed	4.30 (3.15–5.45)	1.63 (1.02–2.59)	0.92 (0.50–1.69)	1.20 (0.71–2.02)
Occupational skill level				
I	3.00 (2.33–3.67)	3.50 (2.14–5.73)	3.43 (1.89–6.23)	Excluded from the analysis
II	4.04 (3.64–4.45)	2.42 (1.53–3.84)	2.48 (1.48–4.12)	Excluded from the analysis
III	4.78 (3.93–5.67)	1.72 (0.98–3.03)	1.87 (1.00–3.46)	Excluded from the analysis
IV	6.39 (5.54–7.23)	1.00 (reference)	1.00 (reference)	Excluded from the analysis
I-II (aggregated)	3.79 (3.44–4.14)	1.98 (1.47–2.68)	Excluded from the analysis	1.85 (1.32–2.61)
III-IV (aggregated)	5.64 (5.00–6.28)	1.00 (reference)	Excluded from the analysis	1.00 (reference)
**Aetiologies**				
Alcohol	4.10 (3.67–4.53)	2.83 (1.39–5.76)	1.63 (0.68–3.90)	1.71 (0.72–4.09)
Cryptogenic	1.80 (1.24–2.36)	6.67 (3.20–13.9)	2.89 (1.17–7.13)	3.01 (1.22–7.43)
Hepatitis C	5.32 (4.48–6.15)	1.81 (0.84–3.88)	1.97 (0.77–5.03)	2.16 (0.85–5.49)
PBC	6.40 (5.23–7.57)	1.00 (reference)	1.00 (reference)	1.00 (reference)
NAFLD	2.80 (1.95–3.65)	3.87 (1.70–8.80)	2.55 (0.95–6.86)	2.74 (1.02–7.36)
AIH	5.41 (3.99–6.84)	1.77 (0.73–4.28)	1.96 (0.70–5.50)	2.01 (0.72–5.60)
Other causes	4.74 (3.49–5.98)	2.09 (0.90–4.89)	2.20 (0.82–5.92)	2.51 (0.94–6.71)
**Severity**^**a**^				
MELD				
MELD < 10	5.60 (5.09–6.11)	1.00 (reference)	1.00 (reference)	1.00 (reference)
MELD 10–14	4.21 (3.57–4.85)	1.66 (1.22–2.26)	1.25 (0.88–1.77)	1.28 (0.90–1.61)
MELD ≥15	2.87 (2.40–3.34)	2.69 (2.06–3.50)	1.05 (0.73–1.52)	1.08 (0.74–1.54)
Child-Pugh class				
A	6.46 (5.99–6.92)	1.00 (reference)	1.00 (reference)	1.00 (reference)
B	3.24 (2.77–3.71)	3.41 (2.55–4.55)	2.35 (1.68–3.29)	2.38 (1.70–3.34)
C	2.19 (1.64–2.76)	5.37 (3.91–7.36)	5.75 (3.67–9.02)	5.93 (3.78–9.31)

*AIH* Autoimmune hepatitis; *CI* Confidence Interval; *HCC* Hepatocellular carcinoma; *MELD* Model for end-stage liver disease; *NAFLD* Non-alcoholic fatty liver disease; *PBC* Primary biliary cholangitis. Patients were followed up until December 31st 2019. Cox regression models were used to calculate hazard ratios for death or transplantation, where each variable was adjusted for all the other variables presented in this table, in addition to adjustments for comorbidity (arterial hypertension, cardiac artery disease, diabetes mellitus, and obesity). In a separate Cox regression model, the variable “occupational skill level” (tretrachotomous) was substituted by a dichotomous variable representing the aggregated occupational skill levels I-II and III-IV. This substitution did not meaningfully changed the calculated hazard ratios for marital and employment status

^a^ MELD-score and Child-Pugh class calculated for 95.5% of patients

The mean survival in occupational skill level IV was significantly greater than the mean survival for occupational skill level I and II (6.39 vs 3.00 and 4.04 years, respectively; *p* < 0.0001) (Table 4). The calculated HRs in univariate analysis were consistent with these findings (*p* < 0.0001) (Fig. 2). Occupational skill level was also a strong predictor of mortality in the multivariate analysis (Table 4). When aggregated, occupational skill levels I-II also had poorer mean survival (3.79 vs 5.64 years; *p* < 0.0001) and higher HR compared to the aggregated occupational skill levels III-IV (Fig. 2). This association was also significant in multivariate analysis (Table 4).

Occupational skill level and Child-Pugh at cirrhosis diagnosis were statistically significant predictors of mortality in multivariate analysis (Table 4). Since there were statistically significant differences regarding the prevalence of a given Child-Pugh class between the different occupational skill levels (Table 3), we examined the interaction between the two variables in the Cox models. There were no statistically significant interactions between occupational skill level and Child-Pugh, neither in the original model (occupational skill level I through IV; *p* = 0.381) nor in the modified one (aggregated occupational skill level I-II and III-IV; *p* = 0.530). Likewise, there were no statistically significant interactions between occupational skill level and MELD-score in the original or in the modified Cox model (*p* = 0.334 and 0.483; respectively).

**Fig. 3:**
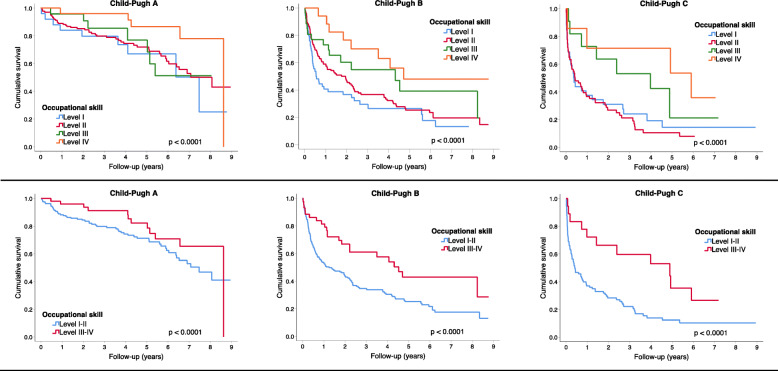
Kaplan-Meier survival curves in the cohort of 582 patients with cirrhosis diagnosed in Halland (2011–2018). Compared by occupational skill level and stratified by Child-Pugh class at cirrhosis diagnosis. Occupational skill level I through IV (above) and I-II vs III-IV (bellow). Occupational skill levels according to the International Standard Classification of Occupations, defined by the International Labour Organisation in 2008 (http://www.ilo.org)

Upon a given Child-Pugh class, occupational skill level was not associated with mean survival among patients with Child-Pugh A (Fig. 3). However, there were distinct associations between mean survival and occupational skill level among patients with Child-Pugh B and C (Fig. 3). These associations were more evident when the aggregated occupational skill levels (I-II vs III-IV) were compared upon the observed Child-Pugh class att cirrhosis diagnosis (Fig. 3).

### Liver transplantation

A total of 18 patients had undergone a transplant and the median age at transplantation was 58 years (range 37–72). The median time from diagnosis to transplantation was 1.75 years (range 0.08–5.40). There were no statistically significant differences among these patients with regard to sex (10 women; *p* = 0.093), aetiology (*p* = 0.340), marital status (13 married; *p* = 0.170) or occupational skill level (*p* = 0.065). However, most transplant patients (72%; *p* < 0.0001) were employed at the time of their transplant surgery.

### Causes of death

Among the 319 deceased patients, most died due to acute on chronic liver failure and related complications (42%). HCC was the second common cause of death (21%), followed by infection (12%), heart disease (8%), and other cancer forms (8%). Other causes of death, such as stroke, lung or kidney failure, accounted for 9% of all deaths. There were no statistically significant differences regarding the cause of death upon sex, marital status, employment status, or occupational skill level.

## Discussion

In this population-based study of 582 cirrhosis patients, we found strong associations between occupational skill level and transplant-free mean survival and, correspondingly, between occupational skill level and mortality. We found large mortality-related inequities between different occupational skill levels, especially between the lower and the higher levels. Married patients had greater mean survival and lower HR for death or transplantation when compared to previously married. However, marital status was not a statistically significant predictor for mortality in multivariate analyses. Similar associations were observed for employment status. Employed showed the best mean survival and lowest HR in univariate analyses. Nevertheless, employment status failed as a predictor of mortality in multivariate analyses. Neither marital or employment status nor occupational skill level were associated with the registered causes of death.

The associations between SES and survival in cirrhosis patients have been comprehensively described for patients in the US [20, 21]. However, only a few studies have described the associations between SES and cirrhosis survival in European populations [15, 16]. In a nationwide study from Denmark, a total of 1765 patients with cirrhosis diagnosed between 1999 and 2001 were followed-up until December 31st 2003 [15]. The main outcome was survival time and the authors reported better survival among married and unmarried patients when compared to divorced patients [15]. Disability retired had poorer survival compared to employed and unemployed [15]. Personal income level was not associated with overall survival [15]. This study did not investigate the associations between occupation and survival.

There are some fundamental differences regarding the epidemiology of cirrhosis between Denmark and Sweden. Alcohol-related cirrhosis is less often seen in Sweden (49–51%) compared to in Denmark (69–79%) [4–6, 34, 35]. Additionally, the median age at cirrhosis diagnosis is higher in Sweden (60–69 vs 56 years) [4–6, 34, 35]. Importantly, only 20% of the included patients in Danish studies were older than 60 years at cirrhosis diagnosis [35]. Given the high age at cirrhosis diagnosis in Sweden, employment status might be a less suitable SES indicator. Nevertheless, cirrhosis among the unemployed was associated with more severe disease at diagnosis when compared to employed patients. Employed patients were also youngest at diagnosis and received most of the transplanted organs. These differences might be related to income level, which in Sweden is generally higher among employed compared to unemployed.

The Swedish healthcare system is mainly tax-based and aims to ensure everyone has equal access to healthcare services [22]. Personal income level appears to be of minor importance for the accessibility to healthcare for the general population in Sweden [22]. Economic differences have still been reported to be the strongest indicator of SES in studies of health inequalities in old age [36]. In the absence of economic data, we chose to examine the importance of occupational skill level. Occupation has been reported as an alternative SES indicator, comparable to educational level or social class [36]. Occupational skill level in Sweden might be related to educational level. Most patients in the highest occupational skill levels were either employed or pensioners, which also have higher mean disposable income. Additionally, occupational skill level is strongly associated with average monthly salary per se. It is then plausible that the associations between mean survival, HR and occupational skill level, reported by us, are to a high extent mediated by a combination of higher education, better employment status, more stable marital status, and higher income level.

We have shown that lower occupational skill level is associated with more severe cirrhosis at diagnosis. The prevalence of Child-Pugh C gradually increased from the highest occupational skill level through the lowest. A similar pattern was observed among the observed complications, although only ascites and hepatic encephalopathy were statistically significant. Importantly, the two highest occupational skill levels were characterised by (mostly) married individuals, who were either employed or pensioners at the time of cirrhosis diagnosis. This might indicate social and economic stability, which is in contrast with the associations observed in occupational skill level I. The latter being characterised by (mostly) never- or previously married, and disability retired or unemployed.

Child-Pugh and MELD score are arguably the most widely used score systems for the assessment of prognosis in liver cirrhosis [30, 31]. Their prognostic values have been reviewed before [37–39]. Despite having minor differences in some specific conditions, both score systems appear to have similar prognostic values in most cases [40]. Our results further validate prior observations as Child-Pugh and MELD score were both associated with mean survival. We have further shown the influence of occupational skill level among patients with a given Child-Pugh class, regarding HR and mean survival. For instance, patients with Child-Pugh C, which already have a high unadjusted HR for death or transplantation, might have a considerably higher HR depending of their occupational skill level. While Child-Pugh C might have a HR of 5.75 among patients with occupational skill level IV (5.75 × 1.00), the corresponding HR among patients with occupational skill level I might be 3.4 times higher (19.7 = 5.75 × 3.43).

The implementation of SES-related variables in current score systems, such as MELD or Child-Pugh, is not easily achieved and further research is needed. However, our results and previous observations, indicate that clinicians should considerer SES as an important factor when assessing mortality risk by validated score systems, especially among patients with decompensated cirrhosis.

The findings from our study should be interpreted with some limitations in mind. As previously described, marital status, employment status, and occupation were extracted from medical journals, meaning that the data were patient self-reported. There were some occupational variations between skill level groups, but individual patients in one occupational skill level often reported different occupations within the same skill level category. Nevertheless, it is possible that some patients were misplaced regarding their marital status, employment status, or main occupation.

Although occupational skill level is very likely to be associated with personal income and/or educational level, we must emphasise that our results can neither confirm nor deny the associations between personal income and/or educational level and cirrhosis survival reported by others, as we did not have access to individual data regarding personal income and educational level. Similarly, we did not have access to household income level or occupational skill level for a patient’s first-degree relatives. High SES among first-degree relatives could theoretically act as a protective factor, independently of a patient’s own SES.

The incidence of NAFLD-related cirrhosis in Sweden seems to be low [5]. However, we believe that the majority of patients with cryptogenic cirrhosis in our cohort had NAFLD instead. We have previously discussed the subject elsewhere [6], and further analysis of the similarities between NAFLD and cryptogenic cirrhosis is beyond the scope of this manuscript.

Clinical examination-based health measures and the high accessibility to reliable patient and follow-up data are important strengths of the present study.

Additional population-based studies are needed to confirm our findings and investigate further the associations between SES and cirrhosis mortality, especially in other geographical areas in Europe. Prospective studies are also needed to study the potential effect of SES on mortality risk in cirrhosis patients.

## Conclusions

Occupational skill level was strongly associated with mean survival and mortality risk. Inequities in survival might involve several indicators of SES, of which occupation appears to be of noticeable importance. The poorer prognosis among patients in lower occupational skill level groups could not be explained by differences in sex, age, marital status, employment status, cirrhosis severity, or comorbidity. There were no significant differences regarding the cause of death upon marital status, employment status, or occupational skill level.

Even in the existence of a universally provided, and highly available healthcare system, lower SES (defined as lower occupational skill level), was strongly associated with more severe disease at diagnosis and poorer survival. Our results further emphasise the importance of health promotion and liver disease prevention among the most vulnerable groups in society.

## Supplementary Information

**Additional file 1: Supplementary Material 1**. Diagnoses (ICD-10-SE) used to identify possible patients with cirrhosis in Halland (Sweden), 2011–2018. Contains the ICD-10-SE codes used for the identification of the study cohort.

**Additional file 2: Supplementary Material 2**. Relationship between employment status and equivalised annual disposable income (above), and between occupational skill level and average monthly salary (below). Differences in annual disposable income and average monthly salary for the Swedish population, by employment status and by occupational skill level, respectively.

**Additional file 3: Supplementary Material 3**. Baseline characteristics of the 582 patients, at the time of cirrhosis diagnosis in Halland, 2011–2018. Relationship between marital status, employment status and occupational skill level.

## Data Availability

The datasets generated and/or analysed during the current study are not publicly available due to legal and ethical restrictions but anonymised datasets are available from the corresponding author on reasonable request.

## Abbreviations

BMI: Body mass index
CI: Confidence intervals
HCC: Hepatocellular carcinoma
HCV: Hepatitis C
HR: Hazard ratios
ICD-10-SE: International classification of diseases, tenth revision – Swedish edition
ISCO-08: International standard classification of occupations 2008
NAFLD: Non-alcoholic fatty liver disease
MELD: Model for end-stage liver disease
SES: Socioeconomic status
SNOMED: Systemized nomenclature of medicine
