# Supplementary material for: Socioeconomic and marital status among liver cirrhosis patients and associations with mortality: a population-based cohort study in Sweden
Source: BMC Public Health. 2020 Nov 30;20:1820. doi: 10.1186/s12889-020-09783-2 (PMC7706059; doi:10.1186/s12889-020-09783-2)
Supplement: Supplementary file 2 — Additional file 2: Supplementary Material 2. Relationship between employment status and equivalised annual disposable income (above), and between occupational skill level and average monthly salary (below). Differences in annual disposable income and average monthly salary for the Swedish population, by employment status and by occupational skill level, respectively. [file 12889_2020_9783_MOESM2_ESM.docx]

|  |
| --- |
|  |
| **Supplementary Material 2.** Relationship between employment status and equivalised annual disposable income (above), and between occupational skill level and average monthly salary (below).  SEK: Swedish krona. SSYK 2012: Swedish Standard Classification of Occupations 2012. SSYK 2012 is based on the International Standard Classification of Occupations 2008 (ISCO-08), which grades occupations into four main occupational skill levels (level I-IV)  Disposable income is the sum of all taxable and tax-free income minus taxes and negative transfers. Incomes from capital gains/losses, that is, the gain/loss arising from a sale (realization) of assets, for example, stocks, mutual funds or real estate are included. Incomes are equivalised, which means that the household disposable income is adjusted for household size by using a weight system. The unit of measurement is equivalised disposable income for persons.  Employed: not classified as students, whose wage and business income exceeds two price basic amounts and is greater than half of the total income from employment and business. Unemployed: not classified as students, whose income from labour market assistance is greater than half of the total income from employment and business. Sick: not classed as students, whose sickness compensation is greater than half of the total income from employment and business (includes people with sickness and activity compensation). Pensioner: not classified as students, whose income from retirement pension, occupational pension, private pension etc. is more than half of the total income from employment and business.  All data extracted from Statistics Sweden (www.scb.se). |
