# Supplementary material for: Socioeconomic and marital status among liver cirrhosis patients and associations with mortality: a population-based cohort study in Sweden
Source: BMC Public Health. 2020 Nov 30;20:1820. doi: 10.1186/s12889-020-09783-2 (PMC7706059; doi:10.1186/s12889-020-09783-2)
Supplement: Supplementary file 3 — Additional file 3: Supplementary Material 3. Baseline characteristics of the 582 patients, at the time of cirrhosis diagnosis in Halland, 2011–2018. Relationship between marital status, employment status and occupational skill level. [file 12889_2020_9783_MOESM3_ESM.docx]

| **Supplementary Material 3.** Baseline characteristics of the 582 patients, at the time of cirrhosis diagnosis in Halland, 2011-2018 | | | | | | | | | | | | |
| --- | --- | --- | --- | --- | --- | --- | --- | --- | --- | --- | --- | --- |
|  | **Marital status, n (%)** | | | **Employment status, n (%)** | | | | **Occupational skill level, n (%)** | | | | **Total, n (%)** |
|  | Married  339 (58) | Never married  141 (24) | Prev. married  102 (18) | Employed  171 (29) | Pensioner  273 (47) | Disability retired  93 (16) | Unemployed  45 (8) | I  115 (20) | II  348 (60) | III  64 (11) | IV  55 (9) | 582 (100) |
| **Socioeconomic and marital status** | | | | | | | | | | | | |
| Marital status |  |  |  |  |  |  |  |  |  |  |  |  |
| Married | - | - | - | 116 (68) | 161 (59) | 41 (44) | 21 (47) | 46 (40) | 208 (60) | 43 (67) | 42 (76) | 339 (58) |
| Never married | - | - | - | 37 (22) | 52 (19) | 34 (37) | 18 (40) | 38 (33) | 86 (25) | 9 (14) | 8 (15) | 141 (21) |
| Previously married | - | - | - | 18 (10) | 60 (22) | 18 (19) | 6 (13) | 31 (27) | 54 (15) | 12 (19) | 5 (9) | 102 (18) |
| Employment status |  |  |  |  |  |  |  |  |  |  |  |  |
| Employed | 116 (34) | 37 (26) | 18 (18) | - | - | - | - | 2 (2) | 116 (33) | 26 (41) | 27 (49) | 171 (29) |
| Pensioner | 161 (48) | 52 (37) | 60 (59) | - | - | - | - | 38 (33) | 175 (50) | 34 (53) | 26 (47) | 273 (47) |
| Disability retired | 41 (12) | 34 (24) | 18 (18) | - | - | - | - | 30 (26) | 57 (16) | 4 (6) | 2 (4) | 93 (16) |
| Unemployed | 21 (6) | 18 (13) | 6 (6) | - | - | - | - | 45 (39) | 0 (0) | 0 (0) | 0 (0) | 45 (8) |
| Occupational skill level |  |  |  |  |  |  |  |  |  |  |  |  |
| I | 46 (14) | 38 (27) | 31 (30) | 2 (1) | 38 (14) | 30 (32) | 45 (100) | - | - | - | - | 115 (20) |
| II | 208 (61) | 86 (61) | 54 (53) | 116 (68) | 175 (64) | 57 (61) | 0 (0) | - | - | - | - | 348 (60) |
| III | 43 (13) | 9 (6) | 12 (12) | 26 (15) | 34 (13) | 4 (4) | 0 (0) | - | - | - | - | 64 (11) |
| IV | 42 (12) | 8 (6) | 5 (5) | 27 (16) | 26 (9) | 2 (2) | 0 (0) | - | - | - | - | 55 (9) |
| Occupational skill level according to the Swedish Standard Classification of Occupations 2012 (SSYK 2012), which is based on the International Standard Classification of Occupations 2008 (ISCO-08). A more detailed description of the SSYK 2012 can be found at Statistics Sweden ([www.scb.se](http://www.scb.se)). All shown comparisons have a p-value < 0.0001. | | | | | | | | | | | | |
